# Health and quality of life differ between community living older people with and without remaining teeth who recently received formal home care: a cross sectional study

**DOI:** 10.1007/s00784-018-2360-y

**Published:** 2018-02-15

**Authors:** A. R. Hoeksema, L. L. Peters, G. M. Raghoebar, H. J. A. Meijer, A. Vissink, A. Visser

**Affiliations:** 1 Department of Oral and Maxillofacial Surgery, University of Groningen, University Medical Center Groningen, PO Box 30.001, NL-9700 RB Groningen, The Netherlands; 2 Department of Oral and Maxillofacial Surgery, and Department of Fixed and Removable Prosthodontics, Dental School, University of Groningen, University Medical Center Groningen, Groningen, The Netherlands

**Keywords:** Community-living older people, Oral health, Dentate, Complete denture, Formal home care, Quality of life

## Abstract

**Objective:**

To assess oral health, health, and quality of life (QoL) of care-dependent community-living older people with and without remaining teeth who recently received formal home care.

**Materials and methods:**

For this cross-sectional observational study, community-living older people (≥ 65 years), who recently (< 6 months) received formal home care, were interviewed with validated questionnaires and underwent an oral examination. Oral health, general health, medicines usage, frailty (Groningen Frailty Indicator), cognition (Minimal Mental State Examination), QoL (RAND 36), and oral health-related QoL (Oral Health Impact Profile-14) were assessed.

**Results:**

One hundred three out of 275 consecutive eligible older people (median age 79 [IQR (Inter Quartile Range) 72–85 years] participated in the study. Thirty-nine patients had remaining teeth and 64 were edentulous. Compared with edentulous older people, older people with remaining teeth scored significantly better on frailty, QoL, physical functioning, and general health. No significant differences were seen in cognition. Dental and periodontal problems were seen in more than half of the patients with remaining teeth. Two third of the edentulous patients did not visit their dentist regularly or at all.

**Conclusions:**

Care-dependent home-dwelling older people with remaining teeth generally were less frail, scored better on physical functioning and general health and had better QoL than edentulous older people. Dental and periodontal problems were seen in approximately 50% of the elderly.

**Clinical relevance:**

Notwithstanding their common dental problems, frailty, health, and QoL are better in home-dwelling older people with remaining teeth. To maintain this status, we advise not only dentists, but also health care workers and governments, to encourage people to maintain good oral health.

## Introduction

Worldwide, many societies are aging. This also counts for the Netherlands. In 2020, around 30% of the people who live in the northern region of the Netherlands will be 65 years of age or older [1]. The number of older people who are older than 80 years will also increase rapidly (prognosis 2020 > 14%) [2]. A growing number of these older people still have their own teeth [3, 4]. Having your own teeth is in general supposed to give better function, but consequently the risk of infections is potentially rising when compared with edentulous persons. Especially when older people become frail, care-dependent and home-bound, the quality of self-care often declines, particularly oral care [5, 6]. As a result, oral problems such as dental infections and periodontitis next to tooth loss and loss of dental function can occur, which has been presumed to negatively impact general health [7–16].

Many studies have been performed to assess oral health in older people. Indwelling older people in nursing homes is worldwide well described in literature [17, 18]. All studies conclude that oral health is poor in these elderly. Knowledge on oral health of community-living older people is sparse, however. It is not yet known whether the oral health of community-living older people differs from that of older people living in nursing homes. Especially the knowledge on oral health and oral status of older people with declining health who are probably heading for admittance in nursing homes in the next period if their health is getting worse and worse, is not well assessed.

The few studies yet published on oral health of community living older people suggest that many older people face oral health problems [19]. However, these studies did not report specifically on the oral status (own teeth, implant supported overdentures, or edentulous) of these community living older people and neither did these studies associate oral status and/or oral health with frailty, activity of daily living (ADL), quality of life (QoL), and/or general health. Tôrres et al. (2015) [20] systematically reviewed the relationship between components of frailty and poor oral health. They concluded that none of the eligible studies showed whether or not poor oral health increases the likelihood of developing signs of frailty, although the reviewed studies did suggest an association between frailty and oral health. Thus, there is a need for well-designed studies that give better insight in the oral status and oral health of community living older people with a focus on the possible associations between frailty, ADL, QoL, general health, and oral status. The aim of the current cross-sectional observational study was to assess oral health, general health, and QoL of care-dependent community-living older people with or without remaining teeth who recently (< 6 months) received formal home care.

## Methods

### Participants

Between January 2015 and January 2016, a cross-sectional pilot study was conducted among all consecutive eligible community-living older people (≥ 65 years) residing in the northern region of the Netherlands who live at their own home and recently (< 6 months) received formal home care provided by three large home care organizations operating in this region. The three participating home care organizations (located in the towns of Groningen-Haren, Hoogezand, and Winschoten) covered a large area. The personal professional caretakers informed all their new clients (clients ≥ 65 years who subscribed for care within the last 6 months) about the study and asked whether the researchers could contact them for further inquiry and participation. Patients were eligible to participate if they were physically and/or cognitively able to be interviewed and to undergo an oral screening. The caretakers informed us if a potential participant was not able to communicate due to severe dementia or whether they were physically too ill to be interviewed. Participation meant that participants should allow for an extensive structured interview (1 h interview) and an oral examination (see below). For all older people who were asked to participate in this study, data on age, gender, and intensity/type of formal home care were available and used for analysis.

Contact information of the potential participants who were willing to take part in the study was provided by the caretakers to the researchers of the University Medical Center Groningen. Next, the researchers contacted the older people by phone. After having obtained written informed consent, the participants were invited to visit a dental care unit of the department of oral and maxillofacial surgery of the University Medical Center Groningen or, if preferred, they were visited at their own home. An extensive structured interview (see below), followed by an oral examination, was performed by either ARH or AV, both geriatric dentists. These dentists had worked together for over 15 years and were experienced in performing oral examinations in geriatric patients [21]. From all non-participants, demographic information and information on formal home care were known.

The institutional review board of our institution provided a waiver (file number M13.145588), as this observational study was not an experimental study with test subjects as defined in the Medical Research Involving Human Subjects Act. Written informed consent was obtained from all participants and the study was performed in accordance with the Declaration of Helsinki.

### Structured interview and questionnaires

The extensive questionnaire took around 60 min to complete. All data was collected by a personal interview of the participant. The following data were obtained during the interview:

- Demographics (age, gender, partnership (y/n), education level (e.g., only lower school finished, lower and secondary school finished, lower professional education/ higher education);
- General health (physical and psychological morbidity, number of medicines);
- Formal home care (e.g., domestic/house cleaning care, personal care, nursing care);
- Informal care (frequency of care given by friends and relatives)

The following validated questionnaires were used:

1. Groningen Frailty Indicator; Frailty was assessed with the Groningen Frailty Indicator (GFI). This instrument comprises 15 items and measures losses of functions and resources in four domains: physical, cognitive, social, and psychological. Scores range from 0 to 15; a score of 4 and higher indicates moderate to severe frailty [22, 23];
2. Minimal Mental State Examination; Cognitive functioning was assessed with the Minimal Mental State Examination [24]. Scores range from 0 to 30. A score of 25 or lower indicates moderate to severe cognitive impairment [24];
3. RAND-36; Generic health-related quality of life was assessed with the RAND 36-Item Health Survey (RAND-36). This measure includes the following subscales: physical functioning, social functioning, role limitations due to physical health problems, role limitations due to emotional problems, general mental health, vitality, bodily pain, and general health perception [25]. The total score range of all scales is 0 to 100, with higher scores indicating better health;
4. OHIP-14; Oral-health-related quality of life (including oral pain and oral discomfort) was assessed with the Oral Health Impact Profile (OHIP-14). This instrument consists 14 items, range 0–56. A higher score indicates lower oral health-related quality of life [26].

### Oral health examination

First oral status (having remaining teeth or being edentulous and wearing complete dentures) was determined. Older people with remaining teeth were examined for the number of teeth present and the presence of dental plaque, calculus, fractured teeth, caries, periodontal disease (three or more periodontal pockets of ≥ 5 mm). The deepest periodontal pocket measured per element was noted. For presence of plaque, the index according to Mombelli et al. (1983) [27] was used (score 0, no detection of plaque; score 1, plaque can be detected by running a probe across the smooth marginal surface of the implant; score 2, plaque can be seen by the naked eye; score 3, abundance amount of plaque). When more than one tooth was present the highest score per dentition was noted.

The presence of calculus (score 1) or the absence of calculus (score 0) was scored. Probing depth was measured at four sites of each tooth (mesially, labially, distally, lingually) by using a periodontal probe (Merit B, Hu Friedy, Chicago, USA); the distance between the marginal border of the mucosa and the tip of the periodontal probe was scored as the probing depth.

Edentulous older people with complete dentures were examined for the fit (good, acceptable, or poor stability) and appearance (fractured parts, wear, etc.) of the dentures. Additional assessments included oral hygiene and whether the participants still visited the dentist regularly (did they visit the dentist for their yearly dental check-ups)? Oral hygiene was rated as good in the absence of visual plaque (score 0 and 1) poor when thin layers of plaque were seen (score 2) and very poor when layers of plaque were present in or on the teeth or dentures (score 3) according to Mombelli et al. (1983) [27],

### Statistical analyses

Baseline characteristics were reported with descriptive statistics. Differences between participating and non-participating older people on age, gender, and intensity of formal home care were evaluated with Pearson Chi-Square tests and Mann-Whitney test. Median scores, including interquartile ranges (IQR), were calculated for all measurement scores, since the data were not normally distributed. Statistical differences between older people subgroups on oral status that differed on measurement scores were examined with Mann-Whitney tests. A *p* value of ≤ 0.05 was considered statistically significant. Statistical analyses were performed with SPSS Statistics 22.0 (SPSS inc. Chicago, Illinois).

## Results

### Participants

**Fig. 1:**
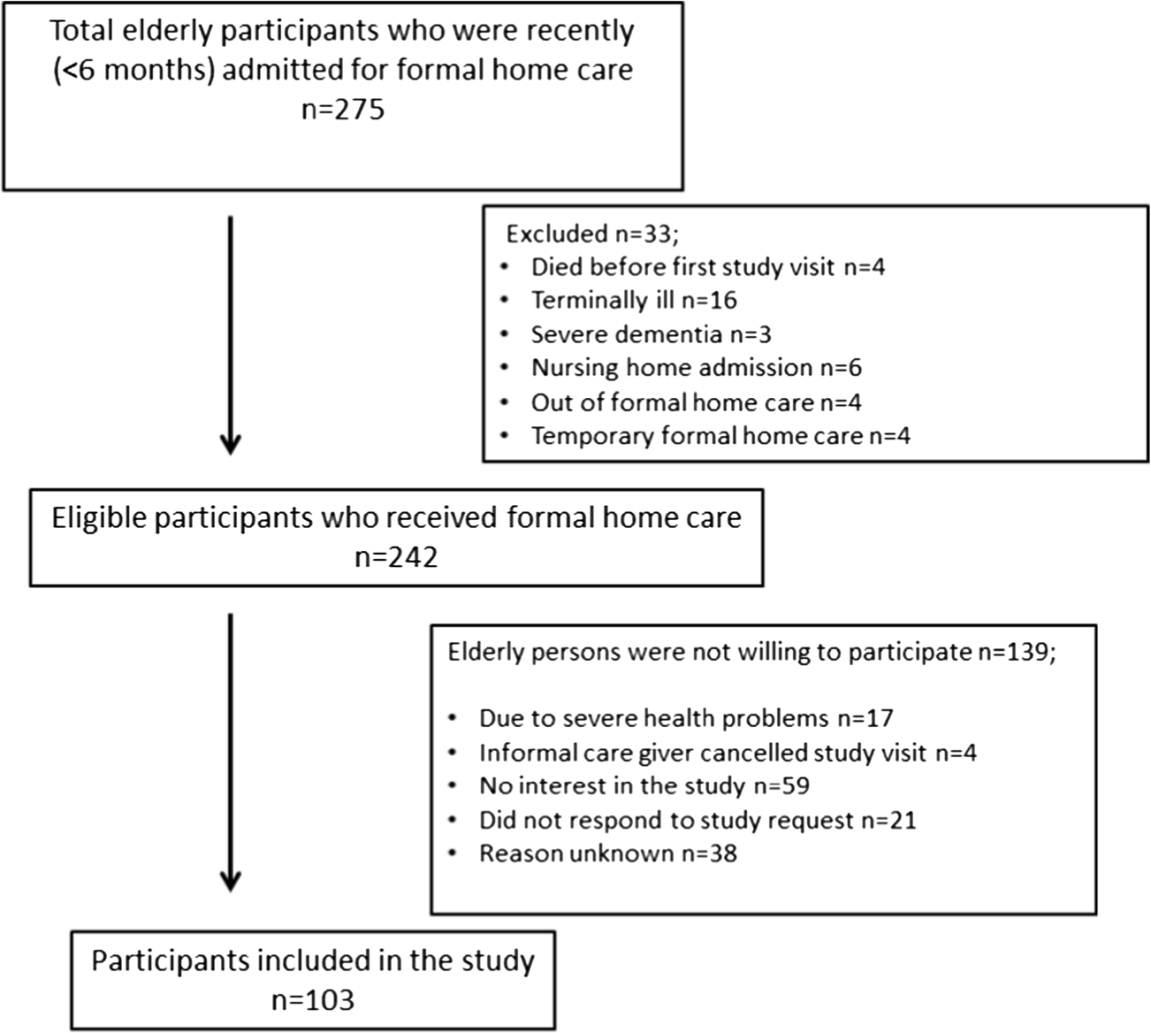
Flow chart of included older people

One hundred three out of a total of 275 consecutive older people who were admitted for formal home care (Fig. 1) participated in this observational study. Professional home caretakers from 33 potential participants asked us not to get in contact with these older people as they were suffering from for instance severe illness (*n* = 16) or dementia (*n* = 3). The main reason of the other older people for not participating in the study was no interest in the study (*n* = 59). Compared with the demographics of non-participating older people, the 103 older people included in the study did not differ on age (*p* = 0.61) and gender (*p* = 0.39). However, non-participating older people received significantly more formal personal care (*p* ≤ 0.001). No information on health problems was available for non-participants.

The 103 consecutive-included participants had a median age of 79 [IQR 72–85] years, and 51% (*n* = 52) of the participants were female (Table 1). Three fourths of the participants reported three or more physical morbidities and one fifth (*n* = 23) at least one psychological morbidity. The median number of medications was 7 [IQR 4–11]. The three most-used medications were anticoagulants (51%), anti-hypertensive’s (44%), and beta blockers (39%). All participants received formal homecare which was mainly for personal care (Table 1). Informal home care was additionally received by 34% (*n* = 35) of the participants (e.g., for assistance on domestic matters, financial, and postal paperwork, etc.).

**Table 1:** Baseline characteristics of the included care dependent community living older people persons

	Total population, *N* = 103	Oral status		
		Remaining teeth, *N* = 39	Edentulous dentures/implants, *N* = 64	*P* value remaining teeth vs edentulous
Demographics				
Age (median, IQR; in years)	79 (72–85)	79 (70–86)	78 (74–84)	0.53
Female (*N*, %)	52 (51)	19 (49)	33 (52)	0.78
Marital status (partner) (*N*, %)	39 (38)	16 (41)	23 (36)	0.61
Education level (*N*, %)				0.09
Primary school or lower	39 (38)	11 (28)	28 (44)	
Secondary school	53 (52)	21 (54)	32 (50)	
Higher education	11 (11)	7 (18)	4 (6)	
Morbidity (*N*, %)				
Physical morbidity^1^				0.02
0–1 disease/disorder	17 (17)	11 (28)	6 (9)	
2 diseases/disorders	10 (10)	5 (13)	5 (8)	
3 diseases/disorders	76 (74)	23 (59)	53 (83)	
Number of medicines (median, IQR)	7 (4–13)	6 (3–9)	8 (4–13)	0.02
Formal care (*N*, %)			0.07	
Domestic/house cleaning care	20 (19)	4 (10)	16 (25)	
Personal care (cloting, bathing)	52 (51)	21 (54)	31 (48)	
Nursing care (medication intake, etc.)	19 (18)	11 (28)	8 (13)	
Domestic, personal, or/and nursing care	12 (12)	3(8)	9(14)	
Lifestyle				
Present smoking	21 (20)	4 (10)	17 (27)	0.05
Alcohol intake				
No alcohol consumption	67 (65)	22 (57)	45 (70)	NA
< 1 day a week	13 (13)	7 (18)	6 (9)	
2–5 days a week	9 (9)	4 (10)	5 (8)	
6–7 days a week	14 (14)	6 (15)	8 (13)	
Informal care (*N*, %)				
Daily informal care by relative/friend	35 (34)	11 (28)	24 (38)	0.32
Oral self-care (*N*, %)				
Poor oral hygiene (plaque score 2 and 3)	55 (53)	23 (61)	32 (50)	0.38
Regular dental visit	32 (31)	26 (67)	6 (9)	≤ 0.001

*NA* not applicable, *IQR* inter quartile range, *N* number

^1^Physical morbidity includes the following diseases: arteriosclerosis, cancer, cerebrovascular disease, coronary heart disease (i.e., angina pectoris, arrhythmia, or myocardial infarction), diabetes mellitus, degenerative neurological disorder (i.e., multiple sclerosis, Parkinson), epilepsy, joint diseases (i.e., rheumatoid) arthritis, kidney failure, muscular diseases, pulmonary diseases (i.e., chronic obstructive pulmonary disease, asthma, dyspnea, emphysema), thyroid disease

^2^Psychological morbidity includes the following: anxiety disorders, dementia, depression

^3^Plaque scores as described in materials and methods

### Oral health examination and self-care

More than half of the participants (63%, *n* = 65) were interviewed and examined at their homes since they experienced difficulties in visiting the dental clinic due to mobility problems (Table 1).This result is in line with the observation that in this study 69% of the participants no longer visited their dentist regularly anymore (Table 1); They only visit the dentist in case of complaints that need to be addressed by a dentist. The oral examination showed that 39 participants had remaining teeth and 64 participants were edentulous and had complete dentures. Of the latter 64 participants, nine participants wore an implant-retained mandibular overdenture.

The oral health status of patients with remaining teeth was poor since caries, fractured teeth, or periodontal disease were common in 77% of the participants (*n* = 30) (Table 2). Fifty percent (*n* = 32) of the edentulous participants had poorly fitting upper dentures, and 30% had poorly fitting lower dentures. In some cases, there were no dentures (*n* = 2) at all (Table 2). There were no clinical signs of peri-implant bone loss in participants with implant-retained mandibular overdentures as the peri-implant sulcus was not deepened in the patients assessed.

**Table 2:** Oral examination outcomes of older people with remaining teeth (*n* = 39) or with complete dentures/implants (*n* = 64)

Eldery with remaining teeth (*N* = 39)	
Number of natural teeth (median, IQR)	18 (11–22)
Caries (*N*, %)	
No caries	18 (46)
1–2 cavities	8 (21)
≥ 3 cavities	13 (33)
Fractured teeth (*N*, %)	
No fractured teeth	22 (56)
1–2 fractured teeth	12 (31)
≥ 3 fractured teeth	5 (13)
Periodontal pockets ≥ 5 mm (*N*, %)	
No pockets	14 (36)
1–2 pockets ≥ 5 mm	3 (8)
≥ 3 pockets ≥ 5 mm	22 (56)
Oral hygiene/plaque scores (*N*/%)	
Good/plaque score 0–1	16 (41)
Moderate plaque score 2	20 (51)
Poor plaque score 3	3 (8)
Older people with complete dentures (*N* = 64)	
Upper jaw denture (*N*, %)	
No denture	2 (3)
Good	8 (13)
Moderate	22 (34)
Poor	32 (50)
Lower jaw denture (*N*, %)	
No denture	5 (8)
Good	10 (16)
Moderate	30 (47)
Poor	19 (30)

### Oral care/self-care

Participants with remaining teeth visited their dentists on regular basis more often than the edentulous participants (67 versus 9%, respectively; Table 1). Reasons for avoiding dental care mentioned were mobility problems (not able to go to the dental office), financial aspects (fear for high costs), disturbed relation with the dental office after change of dental team, cognitive problems (forgot to go or forgot appointments), and dental fear. A large majority of the participants (94%) revealed that they cleaned their teeth by themselves and 89% did not experience any difficulties with this task notwithstanding the poor oral hygiene (plaque scores 2 and 3) that was observed in > 50% of all participants (Table 1).

### Measurement scores

Two thirds of the participants (*n* = 68) were identified as frail (GFI score ≥ 4), and nearly half of them had a mild cognitive impairment (MMSE score between 21 and 26). Participants that differed on oral status scored similarly on cognitive dysfunction (MMSE and GFI cognitive domain, Table 3), but participants with remaining teeth scored significantly better on frailty and QoL (oral health related QoL, physical functioning, general health) than edentulous participants (including those with implant-retained mandibular overdentures, Table 3).

**Table 3:** Median scores (IQR) of cognitive dysfunction, frailty, and (oral health related) quality of life of the total population and older people subgroups who differed on oral status

	Oral status			
Measures	Total population *N* = 103	Remaining teeth *N* = 39	Dentures/implants *N* = 64	*P* value
Cognitive dysfunction (MMSE) ^1^	26 (23–27)	26 (23–27)	26 (23–27)	0.89
Frailty (GFI)^2^	5 (3–7)	3 (2–6)	5 (4–7)	0.01
GFI physical domain	3 (2–5)	2 (1–3)	3 (2–5)	0.02
GFI cognitive domain	0 (0–0)	0 (0–0)	0(0–0)	0.51
GFI psychosocial domain	1 (0–2)	1 (0–2)	1 (1–3)	0.03
Quality of life oral health (OHIP-14)^3^	3 (1–6)	1 (0–4)	4 (2–8)	≤ 0.001
Quality of life (RAND-36)^4^				
Physical functioning	55 (15–80)	61 (20–95)	40 (15–70)	0.05
Social functioning	63 (50–88)	75 (50–100)	63 (50–88)	0.22
Role limitations physical	50 (0–100)	50 (0–100)	100 (0–100)	0.64
Role limitations emotional	100 (33–100)	100 (33–100)	100 (33–100)	0.63
Mental health	76 (60–84)	76 (64–88)	68 (60–84)	0.19
Vitality	60 (40–75)	65 (45–80)	55 (40–74)	0.09
Bodily pain	68 (33–100)	80 (45–100)	61 (33–100)	0.19
General health	55 (35–70)	65 (50–75)	50 (30–70)	0.01

^1^Minimal Mental State Examination

^2^Groningen Frailty Indicator

^3^Oral health impact profile 14

^4^The RAND-36-item Health Survey

## Discussion

This cross-sectional study aimed to assess the oral health, oral status, health and QoL of care-dependent community living older people with and without remaining teeth who recently (< 6 months) received formal home care. Overall oral health of the assessed older people was commonly poor as well as that oral status is associated with frailty, QoL, and general health.

Notwithstanding the dental problems and/or poor oral health in persons with remaining teeth, participants with remaining teeth generally scored better on GFI, Rand-36, and OHIP14 than the edentulous participants. This seems surprising, as poor oral health is assumed to be a health risk [7–16]. The reasons for this apparently paradoxical finding are unclear. A possibility is that oral health of older people with remaining teeth might have been reasonable until they became care dependent in the last few months before the screening. When older people become frail and their general health declines, oral clearance often rapidly reduces, leading to increased risk of oral infections and dental caries [3, 28]. Furthermore, manual skills and cognitive functioning often deteriorate; as result, older people become unable to brush their teeth properly and to visit their dentist regularly [6], or they may simply forget to do so. Another possible explanation is based on previous findings that older people with remaining teeth have a higher socio-economic status and better general health [29–31] which is also the case in this study (Table 1).

It would have been interesting not only to compare dentulous people with edentulous people, as we did in the current study, but also edentulous patients with and without implant-retained overdentures. In the current study, the subgroup of edentulous elderly with an implant-retained overdentures was too small for such analyses. However, in a larger community-based study [31], we could show that frailty was less and quality of life significantly better in elderly with implant-retained mandibular overdentures than in elderly without such a denture. People with a higher education and higher socio-economic status are usually more interested in their own general health and oral health, which may also result in fewer diseases/disorders in later life. This presumption was recently confirmed by Vettore et al. [32] who showed that adults with a higher socio-economic status generally have better oral health.

For this study, we selected older people who were recently referred for formal homecare for the first time. We included only this group and not all older people who received formal home care because the focus of the current study was on the oral health status of older people who were recently admitted to formal home care, thus before health might have declined further. In another study from our group performed in a nursing home [21], we found that even 70% of the older people who are newly admitted to a nursing home had already a poor oral health. In the current study, performed in the same region in the Netherlands, we found poor oral health in approximately 50% of the cases (Table 2). We presume that oral health had declined in the period of sickness before older people were admitted to a nursing home. Based on this presumption, the government and healthcare workers as dentists, doctors, nurses, and even pharmacists should pay attention to the risk of decline of oral health when people’s general health gets worse in order to prevent general health problems caused by poor oral health.

This counts not only for the Netherlands but countries all over the world as the observations made in this study probably will not be representative for just the Netherlands but can probably be generalized for other developed countries. However, oral health in elderly in these other countries might be even worse as the national dental and health care in the Netherlands is on a high standard and affordable for many people.

A possible limitation of our descriptive study is the rather low response rate (approximately 40%) and the number of older persons studied. Such a low-response rate is in line with other studies performed in older people living at their own homes [32–34] as well as those groups of 100 persons for studies like this are also common [16, 35]. Moreover, the problems we encountered in our study were comparable to those reported in the other studie, e.g., when we telephoned the older people initially to inform them about the study, we told them that the participants themselves would benefit from participating in the study as they were offered a free check-up (including additional diagnostics when needed) and free advices concerning their oral health. However, when we asked them whether they were willing to participate in the study, many older people recalled that they had no interest in participation in research at all and that oral care was not on their personal priority list. They did not want to visit a dental office or being visited at home by a dentist. Many of them recalled that their energy level was too low to join any study. As the non-participants needed substantially more formal personal care than the participants, is it likely that their oral health might have been even worse than the oral health of the participating subjects and presumably more resembles the oral health of patients recently admitted to a nursing home [21, 36]. Another reason for not participating might be that some of them were aware of their possibly poor oral health and declined to participate because they were ashamed of this. On the other hand, other older people were keen on participating as they knew that they were in need of dental care but did not know how to get this care or how to pay for it; by participating in the study, they received free dental consultation.

## Conclusion

From this cross-sectional observational study, it can be concluded that care-dependent community-living older people who have their own teeth generally score better in terms of physical functioning, frailty, and general health than edentulous older people. However, in 50% of the participants with remaining teeth, dental and periodontal problems were seen. As older persons with remaining teeth generally perform better, it is advised to dentists as well as healthcare workers and governments to encourage all people, not only elderly, to maintain good oral health and a functional dentition thereby decreasing the risk of general health problems that might arise in a later stage of life.
